# Antibacterial, anti-glucosidase, and antioxidant activities of selected highland ferns of Malaysia

**DOI:** 10.1186/1999-3110-54-55

**Published:** 2013-11-07

**Authors:** Tsun-Thai Chai, Sanmugapriya Elamparuthi, Ann-Li Yong, Yixian Quah, Hean-Chooi Ong, Fai-Chu Wong

**Affiliations:** 1 Centre for Biodiversity Research, Universiti Tunku Abdul Rahman, 31900 Kampar, Malaysia; 2 Department of Chemical Science, Faculty of Science, Universiti Tunku Abdul Rahman, 31900 Kampar, Malaysia; 3 Institute of Biological Sciences, Faculty of Science, University of Malaya, 50603 Kuala Lumpur, Malaysia

**Keywords:** Antibacterial, Anti-glucosidase, Antioxidant, Fern, Flavonoid, Hydroxycinnamic acid, Proanthocyanidin

## Abstract

**Background:**

Ferns contain natural products with potential therapeutic applications. Current knowledge of the pharmacological properties of ferns, specifically those growing at high altitudes, is limited. This study aimed to evaluate the phytochemical contents as well as antibacterial, anti-glucosidase, and antioxidant activities of four highland ferns in Malaysia.

**Results:**

Aqueous extracts of the leaves and rhizomes of *Cyathea latebrosa*, *Dicranopteris curranii*, *Gleichenia truncata*, and *Phymatopteris triloba* were analysed. *P. triloba* leaf extract had the highest contents of total flavonoids (118.6 mg/g dry matter), hydroxycinnamic acids (69.7 mg/g dry matter), and proanthocyanidins (29.4 mg/g dry matter). *P. triloba* leaf and rhizome extracts as well as *G. truncata* leaf extract inhibited the growth of both Gram-positive and Gram-negative bacteria. *P. triloba* leaf extract produced a minimum inhibitory concentration (MIC) value of 0.78 mg dry matter/mL when tested against *Pseudomonas aeruginosa*, which is 2.5-fold higher than that of ampicillin. Among all extracts, *P. triloba* leaf extract had the highest anti-glucosidase activity (EC_50_ = 56 μg dry matter/mL) and also the highest antioxidant potential based on DPPH radical scavenging and Ferric Reducing Antioxidant Power assays. Antioxidant activities of both the leaf and rhizome extracts correlated positively with total flavonoid and hydroxycinnamic acid contents (R^2^ = 0.80–0.95). On the other hand, anti-glucosidase activity correlated with total proanthocyanidin contents in both the leaf and rhizome extracts (R^2^ = 0.62–0.84).

**Conclusions:**

In conclusion, highland ferns are potential sources of antibacterial agents, glucosidase inhibitors, and antioxidants.

**Electronic supplementary material:**

The online version of this article (doi:10.1186/1999-3110-54-55) contains supplementary material, which is available to authorized users.

## Background

Ferns are a rich source of natural products with therapeutic potential. Bioactive constituents of ferns exhibit diverse pharmacological properties, which include antioxidant, antibacterial, anti-tumour, and anti-inflammatory activities (Ho et al.^2010^). While the bioactivities of ferns have been previously investigated, little attention has been given to highland ferns. Plants growing at high altitudes are known to produce increased levels of phenolic compounds and exhibit enhanced antioxidant activity (Spitaler et al.^2008^; Rawat et al.^2011^). Hence, highland ferns may be a rich source of bioactive natural products.

*Cyathea latebrosa* (Family Cyatheaceae), *Dicranopteris curranii* (Family Gleicheniaceae), *Gleichenia truncata* (Family Gleicheniaceae), and *Phymatopteris triloba* (Family Polypodiaceae) are four highland ferns which occur not only in Malaysia, but also the rest of South-east Asia (Holttum^1966^; Piggott^1988^). There is no documentation in the literature of the uses of these four ferns as therapeutic agents. Notably, some species in the genera of *Cyathea*, *Dicranopteris*, *Gleichenia* and *Phymatopteris* are used as traditional remedies for various diseases (Ho et al.^2010^; Su et al.^2011^). Hence, we endeavoured to fill the gaps in current knowledge about the therapeutic potential of *C. latebrosa*, *D. curranii*, *G. truncata* and *P. triloba.*

Current interest in searching for therapeutic agents of plant origin is partially promoted by concerns about the side effects of conventional therapeutic agents. For example, existing glucosidase inhibitors (*e.g.* Acarbose) used in the management of diabetes cause side effects, such as flatulence and diarrhoea (Kumar et al.^2012^). Concerns about the toxicity of synthetic antioxidants are also driving current interest in searching for natural antioxidants (Razab and Aziz^2010^). Moreover, there is an urgent need for finding new antibacterial agents due to the increased incidence of bacterial resistance against conventional antibiotics (Daglia^2012^).

At present, the phytochemical profiles of *C. latebrosa*, *D. curranii*, *G. truncata* and *P. triloba* are unknown. However, previous studies have shown that bioactive constituents of ferns mainly belong to the families of phenolics, terpenoids, and alkaloids (Ho et al.^2010^). Flavonoids, hydroxycinnamic acids, and proanthocyanidins are important classes of health-promoting phenolic phytochemicals (El Gharras^2009^). The antibacterial, anti-glucosidase, antioxidant, and other bioactive effects of these phytochemicals were previously reviewed (Cushnie and Lamb^2005^; El Gharras^2009^; Kumar et al.^2011^). Hence, the goal of our study was two-fold: (1) To evaluate the antibacterial, anti-glucosidase, and antioxidant activities of the leaf and rhizome extracts of *C. latebrosa*, *D. curranii*, *G. truncata*, and *P. triloba*; and (2) to determine whether such activities can be attributed to the contents of flavonoids, hydroxycinnamic acids, and proanthocyanidins in the extracts.

## Methods

### Plant material

Leaf and rhizome samples of four fern species, namely *Cyathea latebrosa* (Wall. ex. Hook) Copel., *Dicranopteris curranii* Copel., *Gleichenia truncata* (Willd.) Spreng., and *Phymatopteris triloba* (Houtt.) Pichi Serm., were collected from Cameron Highlands, Malaysia, in January 2012. Collection site elevation is 1495 m. The species of the ferns were authenticated by H.-C. Ong. Voucher specimens of *C. latebrosa*, *D. curranii*, *G. truncata*, and *P. triloba* (numbered TTC01/2012(1), TTC01/2012(2), TTC01/2012(3), and TTC01/2012(4), respectively) were deposited at the Department of Chemical Science, Universiti Tunku Abdul Rahman, for future reference.

### Preparation of aqueous extracts

The leaf and rhizome samples were cleaned and then oven-dried at 45°C for 72 h. The dried samples were ground to powder using a Waring blender. Extracts were prepared by mixing the pulverised samples with autoclaved deionised water at a 1:20 (dry weight: volume) ratio and then incubating the mixture at 90°C for 60 min (Kumaran and Joel karunakaran^2006^). The extracts were clarified by vacuum-filtration followed by centrifugation at 8600 *g* and 4°C for 10 min. The supernatant obtained, taken as 50 mg dry matter (DM)/mL, was aliquoted (500 μL each) and stored at -20°C until used.

### Determination of total flavonoid, hydroxycinnamic acid, and proanthocyanidin contents

Total flavonoid (TF) content was determined using an aluminium chloride colorimetric assay (Chai and Wong^2012^). TF content was expressed as mg catechin equivalents (CE)/g DM, calculated from a standard curve prepared with 0–300 μg catechin/mL. Total hydroxycinnamic acid (TH) content was determined using the Arnow’s reagent (Matkowski et al.^2008^). TH content was expressed as mg caffeic acid equivalents (CAE)/g DM, calculated from a standard curve prepared with 0–200 μg caffeic acid/mL. Total proanthocyanidin (TPR) content was assessed based on the acid-butanol assay (Porter et al.^1986^). TPR content was calculated with the assumption that effective E^1%, 1 cm, 550 nm^ of leucocyanidin is 460 and expressed as mg leucocyanidin equivalents (LE)/g DM.

### Determination of antibacterial activity

Minimum Inhibitory Concentration (MIC) assay was carried out to determine the lowest extract concentration required to inhibit bacterial growth. The assay was performed based on published protocols (Andrews^2001^; Wiegand et al.^2008^) with slight modifications. Two Gram-positive bacteria (*Staphylococcus aureus* and *Micrococcus luteus*) and two Gram-negative bacteria (*Escherichia coli* and *Pseudomonas aeruginosa*) were used in the assay. Briefly, a bacterial inoculum of 5 × 10^5^ colony-forming unit/mL was prepared in Mueller-Hinton Broth and aliquoted into a 96-well sterile microtiter plate. Plant extract was added into the first row of wells, serially diluted to final concentrations of 50.00, 25.00, 12.50, 6.25, 3.13, 1.56, 0.78, and 0.40 mg/mL. The plate was then sealed and incubated at 37°C for 24 h. Next, 20 μL of *p*-iodonitrotetrazolium chloride (0.4 mg/mL) was added to each well, followed by 30 min of incubation at 37°C. Colour change in each well was monitored visually. The lowest extract concentration that inhibited bacterial growth, indicated by the absence of colour change in the well, was taken as the MIC value. For comparison, the assay was carried out using different concentrations of ampicillin (2.50, 1.25, 0.63, 0.31, 0.16, 0.08, 0.04, and 0.02 mg/mL).

### Determination of glucosidase inhibitory activity

Glucosidase inhibitory activity was assessed as previously described (Sancheti et al.^2011^) with minor modifications. A reaction mixture containing 250 μL of 100 mM potassium phosphate buffer (pH 7.0), 150 μL of 0.5 mM 4-nitrophenyl α-D-glucopyranoside, 50 μL of extract, and 150 μL of α-glucosidase (from *Saccharomyces cerevisiae*; 0.1 unit/mL in 10 mM potassium phosphate buffer, pH 7.0) was incubated at 37°C for 30 min. The reaction was ended by adding 600 μL of 200 mM Na_2_CO_3_. The absorbance reading was taken at 400 nm. A blank was prepared for each measurement by replacing α-glucosidase with 10 mM potassium phosphate buffer. Anti-glucosidase activity (%) was calculated using the following equation:

$$\begin{array}{l} \text{Anti}‒\text{glucosidase}\;\text{activity}\left(\%\right)\\ \;=(1-[A_{\text{sample}}/A_{\text{control}}]\;)\times 100\% \end{array}$$

where A_control_ is the absorbance of control reaction (without extract) and A_sample_ is the absorbance in the presence of an extract. Quercetin, which is an effective α-glucosidase inhibitor *in vitro* and *in vivo* (Jo et al.^2009^; Fontana Pereira et al.^2011^; Kim et al.^2011^), was used as the positive control. EC_50_ value, defined as the concentration of extract or quercetin required to achieve 50% anti-glucosidase activity, was determined using linear regression analysis.

### Determination of 1,1-diphenyl-2-picrylhydrazyl (DPPH) radical scavenging activity

DPPH radical scavenging assay was carried out as previously described (Chai and Wong^2012^). Ascorbic acid was used as the positive control. EC_50_ value, which is the concentration of extract or ascorbic acid required to achieve 50% DPPH scavenging activity, was determined using linear regression analysis.

### Determination of Ferric Reducing Antioxidant Power (FRAP)

Ferric reducing activity of the extracts was determined with the FRAP assay (Benzie and Strain^1996^). The FRAP reagent consisted of acetate buffer (300 mM, pH 3.6), 2,4,6-tripyridyl-s-triazine (10 mM), and FeCl_3_.6H_2_O (20 mM) in a 10:1:1 (v:v:v) ratio. A reaction mixture containing 0.2 mL of extract and 1.2 mL of FRAP reagent was incubated at 37°C for 5 min. Absorbance of the mixture was then read at 593 nm. FRAP values are presented in mM Fe^2+^ equivalents, calculated from a standard curve prepared with 0 to 0.40 mM FeSO_4_.7H_2_O. Ascorbic acid was used as the positive control.

### Data analysis

All experiments were carried out in triplicates and data are reported as mean ± standard errors. Statistical analyses were performed using SAS (Version 9.2). Data were analysed by the ANOVA test and means of significant differences were separated using Fisher’s Least Significant Difference (LSD) test at the 0.05 level of probability. Linear regression and correlation analyses were carried out using Microsoft Office Excel 2003.

## Results

The leaf extract of *P. triloba* had the highest TF, TH, and TPR contents compared with all other extracts (Table 1). TPR content of *P. triloba* leaf extract was 21-fold higher compared with *C. latebrosa* leaf extract. Among the rhizome extracts, *P. triloba* had the highest TF, TH, and TPR contents, whereas *C. latebrosa* had the lowest. TF content of the rhizome extract of *P. triloba* was 14-fold higher than that in *C. latebrosa* rhizome extract.

**Table 1:** **Total flavonoid (TF), hydroxycinnamic acid (TH), and proanthocyanidin (TPR) contents in the leaf and rhizome extracts**

	TF (mg CE/g)	TH (mg CAE/g)	TPR (mg LE/g)
**Leaf extract**			
*C. latebrosa*	101.67 ± 6.52^a^	56.32 ± 0.79^a^	1.43 ± 0.01^a^
*D. curranii*	19.07 ± 0.46^b^	34.53 ± 0.12^b^	16.80 ± 0.11^b^
*G. truncata*	65.41 ± 1.46^c^	52.84 ± 1.27^a^	5.97 ± 0.11^c^
*P. triloba*	118.59 ± 1.60^d^	69.70 ± 3.37^c^	29.37 ± 0.14^d^
**Rhizome extract**			
*C. latebrosa*	5.85 ± 0.58^e^	4.84 ± 0.14^d^	2.53 ± 0.09^e^
*D. curranii*	48.89 ± 0.62^f^	28.46 ± 0.50^e^	17.77 ± 0.10^f^
*G. truncata*	50.30 ± 1.64^f^	30.21 ± 0.49^e^	18.50 ± 0.07^g^
*P. triloba*	82.11 ± 2.58^g^	45.82 ± 0.11^f^	24.88 ± 0.10^h^

Values are presented as mean ± SE (*n* = 3). Values in the same column that are followed by different superscript letters are significantly different (*p* < 0.05), as determined using the Fisher’s LSD test.

Antibacterial assays found that the lowest MIC values were consistently observed for the leaf and rhizome extracts of *P. triloba* (Table 2). Among the leaf extracts, only *P. triloba* and *G. truncata* inhibited the growth of Gram-negative bacteria over the range of extract concentrations tested. When tested on *P. aeruginosa* and *S. aureus*, the leaf extract of *P. triloba* produced MIC values that were 2.5-fold and 39-fold higher compared with ampicillin. Among rhizome extracts, *P. triloba*, *G. truncata*, and *D. curranii* were all inhibitory to Gram-positive bacteria but only *P. triloba* was inhibitory to Gram-negative bacteria. The rhizome extract of *P. triloba* produced MIC values that were 10-fold and 104-fold higher than those of ampicillin when tested on *P. aeruginosa* and *S. aureus*.

**Table 2:** **Minimum inhibitory concentrations (MIC) of fern extracts against Gram-positive bacteria (** ***Staphylococcus aureus*** **;** ***Micrococcus luteus*** **) and Gram-negative bacteria (** ***Escherichia coli; Pseudomonas aeruginosa*** **)**

	MIC value (mg/mL)			
	***S. aureus***	***M. luteus***	***E. coli***	***P. aeruginosa***
**Leaf extract**				
*C. latebrosa*	8.33^a^	> 50	> 50	> 50
*D. curranii*	12.50^b^	12.50^a^	> 50	> 50
*G. truncata*	8.33^a^	12.50^a^	12.50^a^	12.50^a^
*P. triloba*	1.56^c^	1.04^b^	6.25^b^	0.78^b^
**Rhizome extract**				
*C. latebrosa*	> 50	> 50	> 50	> 50
*D. curranii*	6.25^a^	8.33^c^	> 50	> 50
*G. truncata*	5.21^d^	6.25^c^	> 50	> 50
*P. triloba*	4.17^d^	2.60^b^	12.50^a^	3.13^c^
Ampicillin	0.04^e^	0.02^d^	0.02^c^	0.31^d^

Values presented are mean values of three replicates. Values in the same column that are followed by different superscript letters are significantly different (*p* < 0.05), as determined using the Fisher’s LSD test. MIC values > 50 mg/mL were not included in the statistical test.

**Figure 1:**
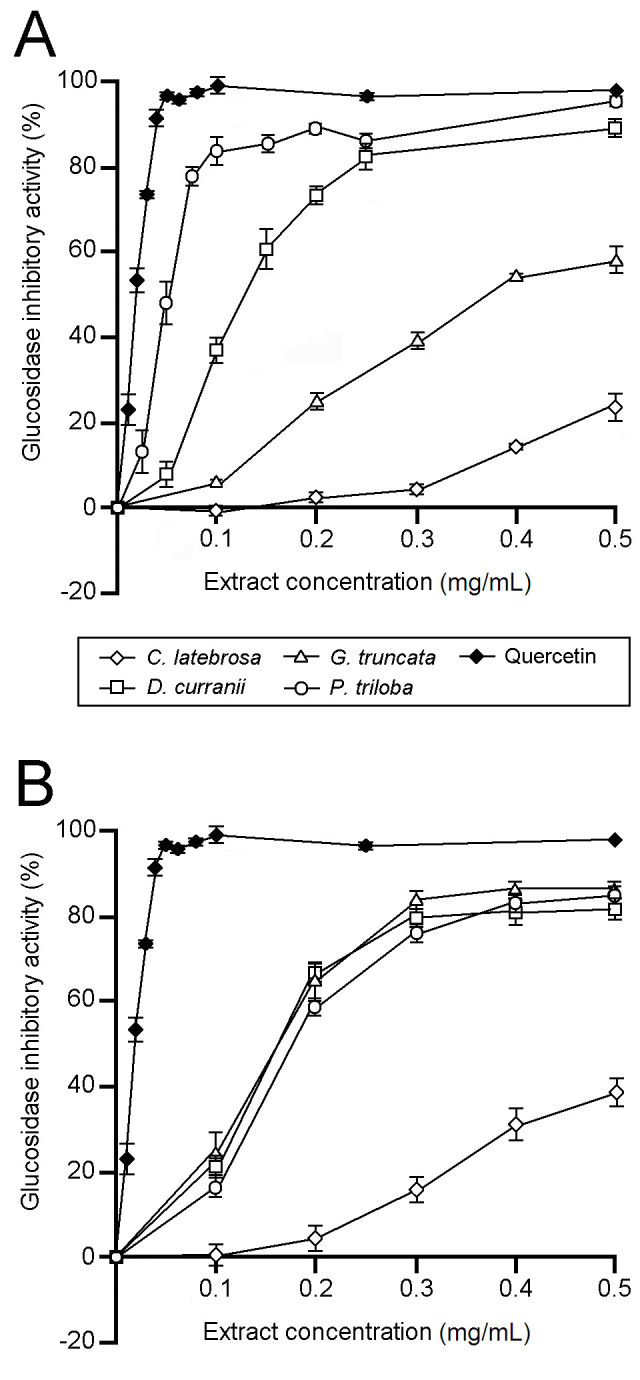
**Glucosidase inhibitory activities of fern extracts. A**, leaf extracts; **B**, rhizome extracts. Data are mean ± SE values (n = 3).

Among the leaf extracts, *P. triloba* had the highest glucosidase inhibitory activity, while *C. latebrosa* had the lowest (Figure 1). The EC_50_ values of the leaf extracts were 56 (*P. triloba*), 143 (*D. curranii*), 408 (*G. truncata*), and 1413 μg DM/mL (*C. latebrosa*). Among the rhizome extracts, *C. latebrosa* had the lowest anti-glucosidase activity, whereas the other three fern species showed similar levels of anti-glucosidase activities. The EC_50_ values of the rhizome extracts were 175 (*G. truncata*), 179 (*D. curranii*), 191 (*P. triloba*), and 755 μg DM/mL (*C. latebrosa*). The EC_50_ value of quercetin was 22 μg/mL.

**Figure 2:**
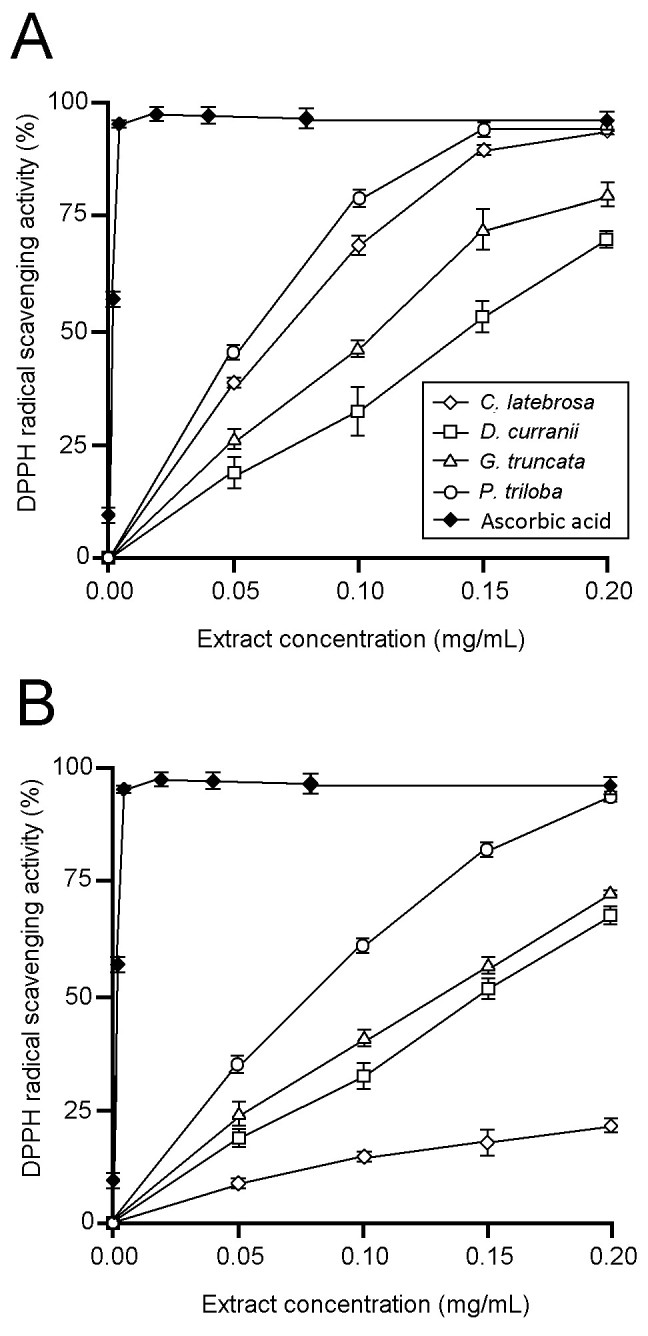
**DPPH radical scavenging activities of fern extracts. A** leaf extracts; **B**, rhizome extracts. Data are mean ± SE values (n = 3).

DPPH scavenging activity of the leaf and rhizome extracts increased in a concentration-dependent manner over the range of concentrations tested (Figure 2). Based on the DPPH scavenging assay, EC_50_ values of the leaf extracts were 73 (*P. triloba*), 79 (*C. latebrosa*), 116 (*G. truncata*), and 144 μg DM/mL (*D. curranii*). There was no significant difference (*p* > 0.05) between the EC_50_ values of *P. triloba* and *C. latebrosa* leaf extracts. EC_50_ values of the rhizome extracts were 97 (*P. triloba*), 133 (*G. truncata*), 148 (*D. curranii*), and 383 μg DM/mL (*C. latebrosa*). There was no significant difference (*p* > 0.05) between the EC_50_ values of *G. truncata* and *D. curranii* rhizome extracts. EC_50_ value of ascorbic acid, the positive control, was 5 μg/mL.

**Figure 3:**
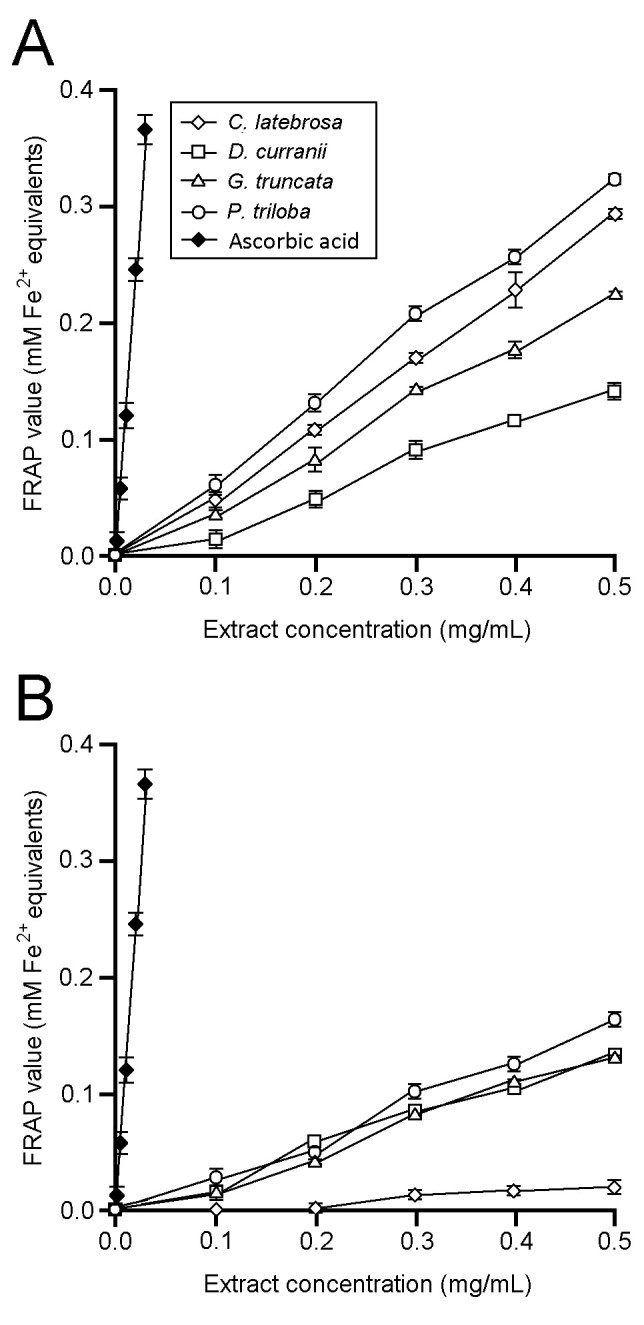
**Ferric Reducing Antioxidant Power (FRAP) values of fern extracts.** **A**, leaf extracts; **B**, rhizome extracts. Data are mean ± SE values (n = 3).

FRAP values of leaf and rhizome extracts increased almost linearly with increasing extract concentrations (Figure 3). The ferric reducing power of leaf and rhizome extracts was lower compared with ascorbic acid. When expressed on the basis of dry mass of plant powder, FRAP values of the leaf extracts in descending order were 696 (*P. triloba*), 571 (*C. latebrosa*), 483 (*G. truncata*), and 305 μmol Fe^2+^ equivalents/g DM (*D. curranii*). FRAP values of the rhizome extracts were 338 (*P. triloba*), 281 (*G. truncata*), 274 (*D. curranii*), and 39 μmol Fe^2+^ equivalents/g DM (*C. latebrosa*). There was no significant difference (*p* > 0.05) between the FRAP values of *G. truncata* and *D. curranii* rhizome extracts.

The strength of correlation between phytochemical contents (TF, TH, and TPR) and the EC_50_ values of anti-glucosidase and DPPH scavenging activities of the fern extracts was analysed. Anti-glucosidase activities of the leaf extracts correlated only with TPR contents (Table 3). The anti-glucosidase activities of the rhizome extracts, by contrast, correlated positively with all three phytochemical parameters (R^2^ = 0.69–0.84). DPPH scavenging activities of leaf extracts correlated strongly and positively with only TF and TH contents (R^2^ = 0.80–0.95). By contrast, DPPH scavenging activities of rhizome extracts correlated strongly with all three phytochemical parameters (R^2^ = 0.84–0.95). FRAP values of leaf extracts only correlated strongly with TF and TH (R^2^ = 0.87–0.91). Strong, positive correlations were found between FRAP values and all three phytochemical parameters in the rhizome extracts (R^2^ = 0.88–0.91).

**Table 3:** **Correlation analyses between phytochemical contents (TF, TH, and TPR) and anti-glucosidase activity, DPPH scavenging activity, and Ferric Reducing Antioxidant Power (FRAP) values of leaf and rhizome extracts**

	Correlation of determination (R^2^)					
Phytochemical contents	EC_50_values for anti-glucosidase activity		EC_50_values for DPPH scavenging activity		FRAP values	
	Leaf extracts	Rhizome extracts	Leaf extracts	Rhizome extracts	Leaf extracts	Rhizome extracts
TF	ns	0.69	0.95	0.84	0.87	0.88
TH	ns	0.73	0.80	0.88	0.91	0.89
TPR	0.62	0.84	ns	0.95	0.55	0.91

Values presented are statistically significant (*p* < 0.05). ns, not statistically significant.

## Discussion

In this study, *P. triloba* leaf and rhizome extracts as well as *G. truncata* leaf extract inhibited the growth of both Gram-positive and Gram-negative bacteria. Notably, when tested against *P. aeruginosa*, comparable MIC values were obtained for *P. triloba* leaf extract and ampicillin. This suggests that *P. triloba* and *G. truncata* are potential sources of broad-spectrum antibacterial agents.

In contrast to Gram-negative bacteria, Gram-positive bacteria were more sensitive to the inhibitory effects of the fern extracts. Similar observations were made in other studies which evaluated the antibacterial efficacy of ferns and other plants (Chew et al.^2009^; Lai et al.^2009^). The insensitivity of Gram-negative bacteria against antibacterial agents is attributable to the permeability barrier posed by the outer membrane of the bacteria and efficient multidrug efflux pumps traversing the bacterial membranes (Delcour^2009^; Li and Nikaido^2009^). Whether bioactive constituents of *P. triloba* and *G. truncata* can compromise these mechanisms in Gram-negative bacteria is currently unclear.

*P. triloba* leaf and rhizome extracts had the highest TF and TPR contents among the fern extracts. Flavonoids may exert antibacterial activity by inhibiting bacterial nucleic acid synthesis, energy metabolism, and cytoplasmic membrane functions (Cushnie and Lamb^2005^). Plant-derived proanthocyanidins may inhibit the growth of pathogenic bacteria by binding strongly to proteins at bacterial cell surfaces (Xu et al.^2012^). Hence, flavonoids, specifically proanthocyanidins, may be key determinants of the antibacterial effects of *P. triloba*.

*P. triloba* leaf extract exhibited the highest glucosidase inhibitory activity among the fern extracts examined. Flavonoids, hydroxycinnamic acids, and proanthocyanidins as well as polyphenol extracts of plant origin are known to inhibit α-glucosidase activity *in vitro* (Hanhineva et al.^2010^). Hence, the high TF, TH, and TPR contents of *P. triloba* leaf extract may partially account for its potent anti-glucosidase activity. Notably, the positive correlation between TPR content and anti-glucosidase activity in both leaf and rhizome extracts suggests that high TPR content may be an indicator of high anti-glucosidase activity in highland ferns.

Our study suggests that the four highland ferns investigated are potential sources of water-soluble antioxidants. *P. triloba* is the most promising source of natural antioxidants among the four fern species. Correlation analyses suggest that flavonoids and hydroxycinnamic acid derivatives may be the key phenolic constituents responsible for the antioxidant activity of the fern extracts. This agrees with the findings of earlier studies on some medicinal plants (Matkowski et al.^2008^; Chai and Wong^2012^). Flavonoids and hydroxycinnamic acids are known to exhibit antioxidant activity (Maurya and Devasagayam^2010^; Procházková et al.^2011^). In our study, TF and TH contents of leaf extracts were generally higher compared with rhizome extracts. This may explain why the leaf extracts showed higher levels of antioxidant activity compared with rhizome extracts. Our findings also imply that high TF and TH contents may be an indicator of high antioxidant activity in ferns and possibly other plants growing at high altitudes.

## Conclusion

In conclusion, aqueous extracts of highland ferns are potential sources of antibacterial agents, glucosidase inhibitors, and antioxidants. Our *in vitro* investigations found that *P. triloba* leaf extract exhibited the highest anti-bacterial, anti-glucosidase, and antioxidant activities among all extracts examined. Phytochemical analyses revealed that *P. triloba* leaf extract had the highest contents of flavonoids, hydroxycinnamic acids, and proanthocyanidins. Correlation analysis suggests antioxidant activities of both the leaf and rhizome extracts may be attributed to their flavonoid and hydroxycinnamic acid contents. On the other hand, anti-glucosidase activity was attributable to total proanthocyanidin contents in both the leaf and rhizome extracts. Optimisation of extraction of bioactive components from *P. triloba*, chemical characterisation of such compounds, and further testing of their effects *in vivo* are warranted in future investigations.

## Authors’ original submitted files for images

Below are the links to the authors’ original submitted files for images. Authors’ original file for figure 1 Authors’ original file for figure 2 Authors’ original file for figure 3

## References

[CR1] Andrews JM (2001). Determination of minimum inhibitory concentrations. J Antimicrob Chemother.

[CR2] Benzie IFF, Strain JJ (1996). The Ferric Reducing Activity of Plasma (FRAP) as a measure of “antioxidant power”: the FRAP assay. Anal Biochem.

[CR3] Chai TT, Wong FC (2012). Whole-plant profiling of total phenolic and flavonoid contents, antioxidant capacity and nitric oxide scavenging capacity of *Turnera subulata*. J Med Plant Res.

[CR4] Chew YL, Goh JK, Lim YY (2009). Assessment of *in vitro* antioxidant capacity and polyphenolic composition of selected medicinal herbs from Leguminosae family in Peninsular Malaysia. Food Chem.

[CR5] Cushnie TPT, Lamb AJ (2005). Antimicrobial activity of flavonoids. Int J Antimicrob Agents.

[CR6] Daglia M (2012). Polyphenols as antimicrobial agents. Curr Opin Biotechnol.

[CR7] Delcour AH (2009). Outer membrane permeability and antibiotic resistance. Biochim Biophys Acta.

[CR8] El Gharras H (2009). Polyphenols: food sources, properties and applications - a review. Int J Food Sci Technol.

[CR9] Fontana Pereira D, Cazarolli LH, Lavado C, Mengatto V, Figueiredo MSRB, Guedes A, Pizzolatti MG, Silva FRMB (2011). Effects of flavonoids on α-glucosidase activity: potential targets for glucose homeostasis. Nutrition.

[CR10] Hanhineva K, Törrönen R, Bondia-Pons I, Pekkinen J, Kolehmainen M, Mykkänen H, Poutanen K (2010). Impact of dietary polyphenols on carbohydrate metabolism. Int J Mol Sci.

[CR11] Ho R, Teai T, Bianchini JP, Lafont R, Raharivelomanana P, Fernández H, Revilla MA, Kumar A (2010). Ferns: from traditional uses to pharmaceutical development, chemical identification of active principles. Working with ferns: issues and applications.

[CR12] Holttum RE (1966). A Revised Flora of Malaya - Volume II Ferns of Malaya.

[CR13] Jo SH, Lee HS, Apostolidis E, Jang HD, Kwon YI (2009). Comparison of antioxidant potential and rat intestinal α-glucosidase inhibitory activities of quercetin, rutin, and isoquercetin. International Journal of Applied Research in Natural Products.

[CR14] Kim SH, Jo SH, Kwon YI, Hwang JK (2011). Effects of onion (*Allium cepa* L.) extract administration on intestinal α-glucosidases activities and spikes in postprandial blood glucose levels in SD rats model. Int J Mol Sci.

[CR15] Kumar S, Narwal S, Kumar V, Prakash O (2011). α-glucosidase inhibitors from plants: a natural approach to treat diabetes. Phcog Rev.

[CR16] Kumar S, Kumar V, Rana M, Kumar D (2012). Enzymes inhibitors from plants: an alternate approach to treat diabetes. Phcog Commn.

[CR17] Kumaran A, Joel karunakaran R (2006). Antioxidant and free radical scavenging activity of an aqueous extract of *Coleus aromaticus*. Food Chem.

[CR18] Lai HY, Lim YY, Tan SP (2009). Antioxidative, tyrosinase inhibiting and antibacterial activities of leaf extracts from medicinal ferns. Biosci Biotechnol Biochem.

[CR19] Li XZ, Nikaido H (2009). Efflux-mediated drug resistance in bacteria: an update. Drugs.

[CR20] Matkowski A, Zielinska S, Oszmianski J, Lamer-Zarawska E (2008). Antioxidant activity of extracts from leaves and roots of *Salvia miltiorrhiza* Bunge, *S. przewalskii* Maxim., and *S. verticillata* L. Bioresour Technol.

[CR21] Maurya DK, Devasagayam TPA (2010). Antioxidant and prooxidant nature of hydroxycinnamic acid derivatives ferulic and caffeic acids. Food Chem Toxicol.

[CR22] Piggott AG (1988). Ferns of Malaysia in Colour.

[CR23] Porter LJ, Hrstich LN, Chan BG (1986). The conversion of procyanidins and prodelphinidins to cyanidin and delphinidin. Phytochemistry.

[CR24] Procházková D, Boušová I, Wilhelmová N (2011). Antioxidant and prooxidant properties of flavonoids. Fitoterapia.

[CR25] Rawat S, Bhatt ID, Rawal RS (2011). Total phenolic compounds and antioxidant potential of Hedychium spicatum Buch. Ham. ex D. Don in west Himalaya, India. J Food Compost Anal.

[CR26] Razab R, Aziz AA (2010). Antioxidants from tropical herbs. Nat Prod Commun.

[CR27] Sancheti S, Lee SH, Lee JE, Seo SY (2011). Screening of Korean medicinal plant extracts for α-glucosidase inhibitory activities. Iran J Pharm Res.

[CR28] Spitaler R, Winkler A, Lins I, Yanar S, Stuppner H, Zidorn C (2008). Altitudinal variation of phenolic contents in flowering heads of *Arnica montana* cv. ARBO: A 3-year comparison. J Chem Ecol.

[CR29] Su W, Li P, Huo L, Wu C, Guo N, Liu L (2011). Phenolic content and antioxidant activity of *Phymatopteris hastata*. J Serb Chem Soc.

[CR30] Wiegand I, Hilpert K, Hancock REW (2008). Agar and broth dilution methods to determine the minimal inhibitory concentration (MIC) of antimicrobial substances. Nat Protoc.

[CR31] Xu Z, Du P, Meiser P, Claus J (2012). Proanthocyanidins: oligomeric structures with unique biochemical properties and great therapeutic promise. Nat Prod Commun.

