# Supplementary figures and images for: Antibacterial, anti-glucosidase, and antioxidant activities of selected highland ferns of Malaysia
Source: Bot Stud. 2013 Nov 7;54:55. doi: 10.1186/1999-3110-54-55 (PMC5430384; doi:10.1186/1999-3110-54-55)

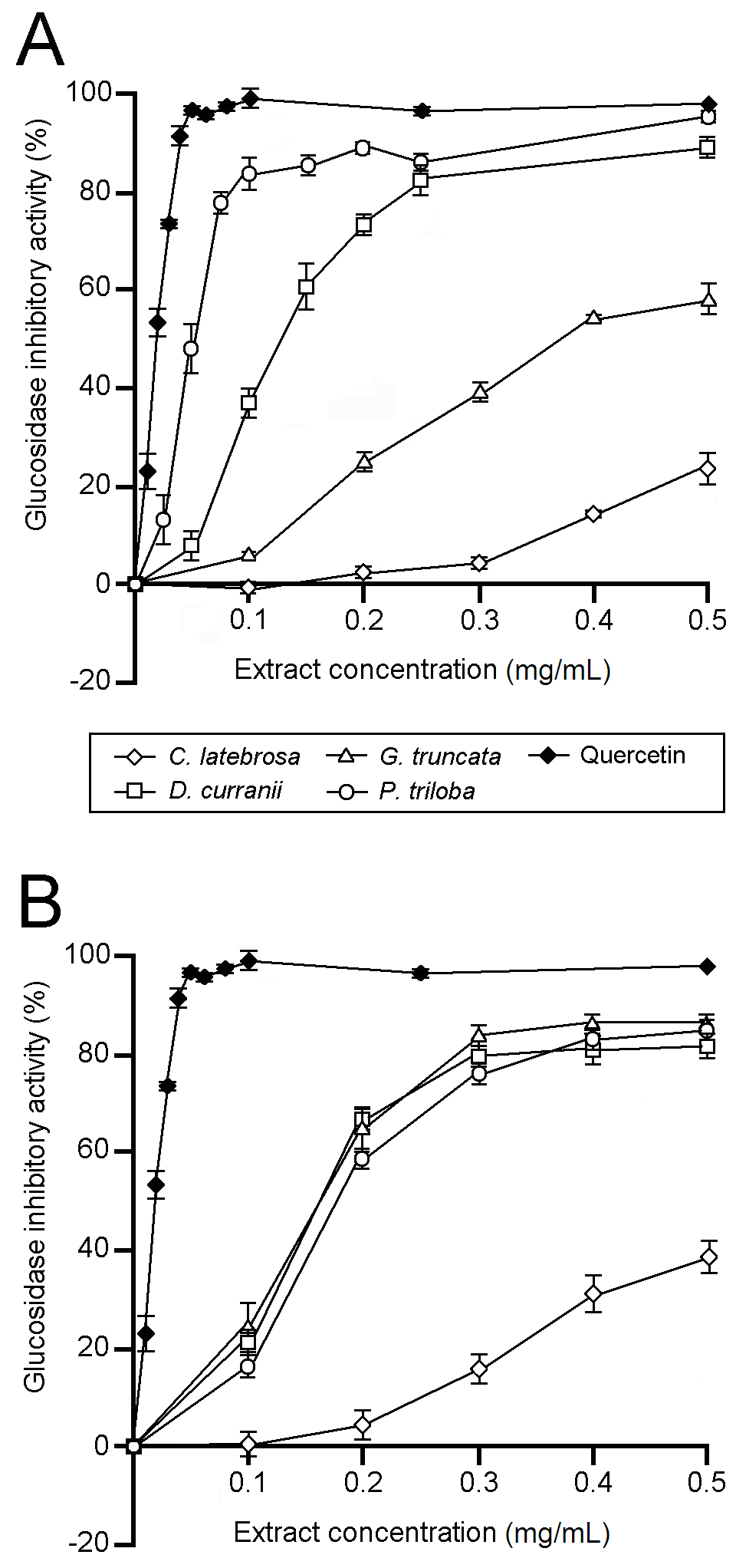

Supplement: Supplementary file 1 — Authors’ original file for figure 1 [file 40529_2013_47_MOESM1_ESM.tiff]

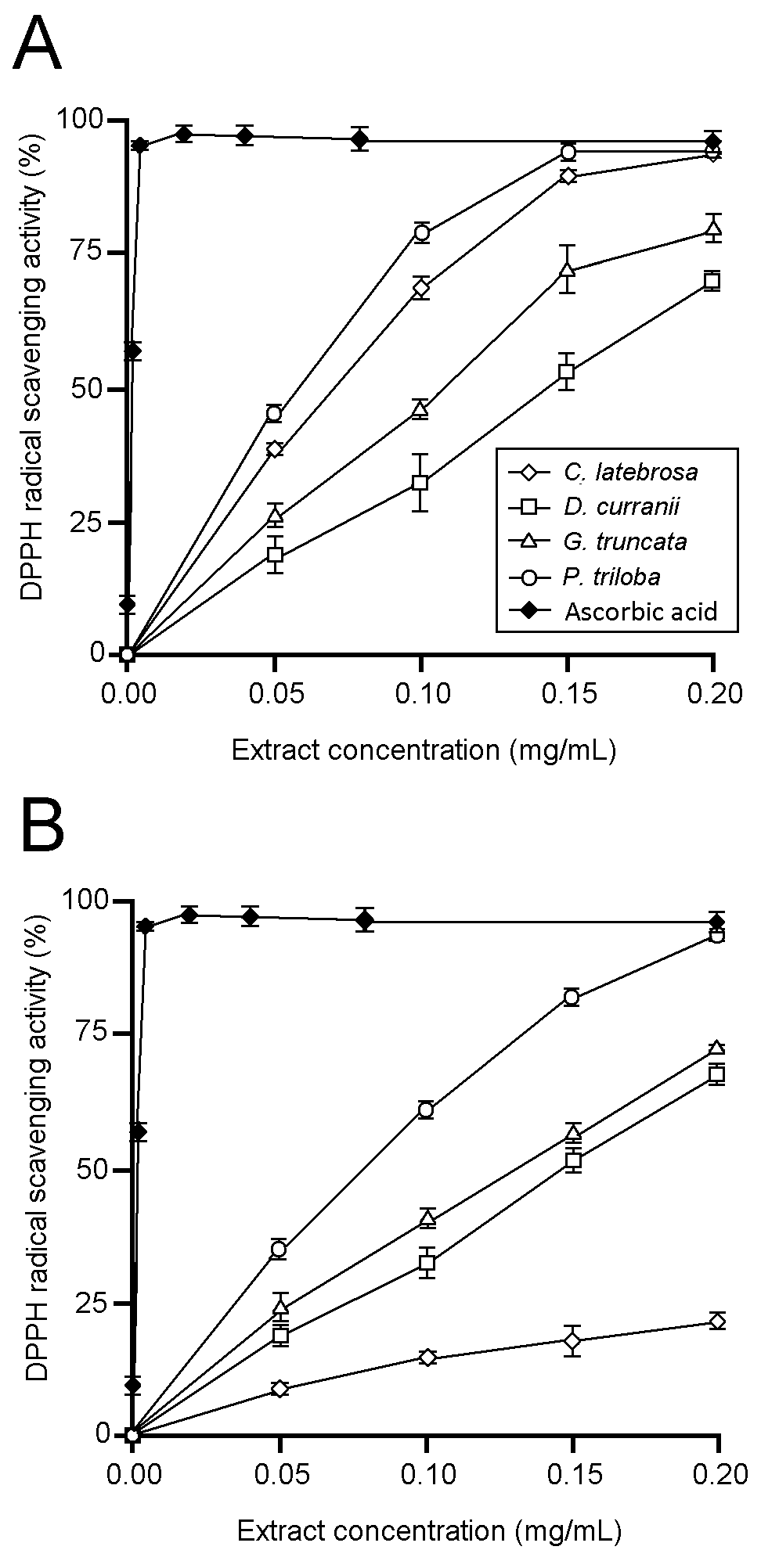

Supplement: Supplementary file 2 — Authors’ original file for figure 2 [file 40529_2013_47_MOESM2_ESM.tiff]

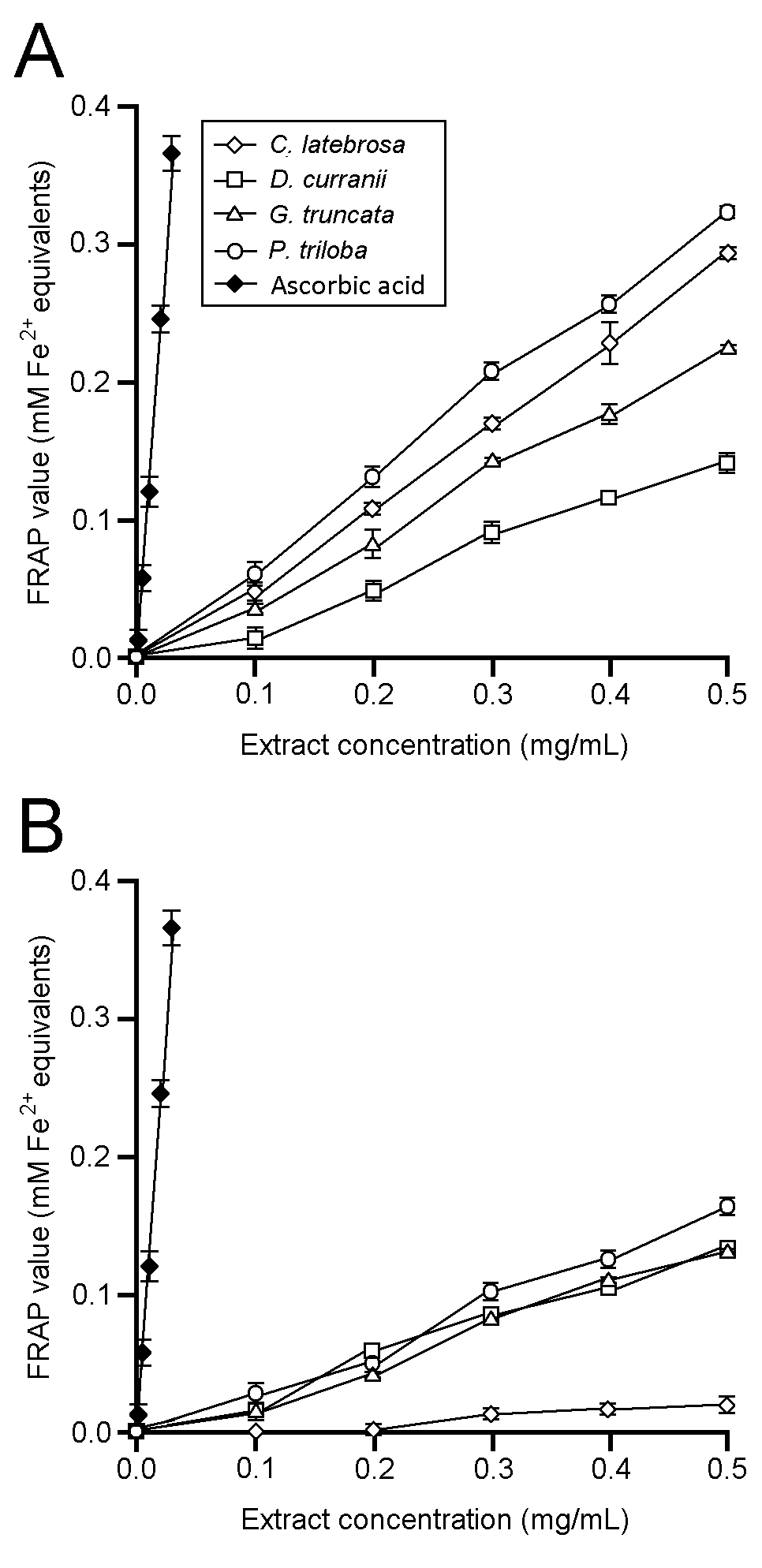

Supplement: Supplementary file 3 — Authors’ original file for figure 3 [file 40529_2013_47_MOESM3_ESM.tiff]
